# Association of Maternal and Fetal Single-Nucleotide Polymorphisms in Metalloproteinase (*MMP1*, *MMP2*, *MMP3*, and *MMP9*) Genes with Preeclampsia

**DOI:** 10.1155/2018/1371425

**Published:** 2018-02-18

**Authors:** Agata Sakowicz, Michalina Lisowska, Lidia Biesiada, Magda Rybak-Krzyszkowska, Agnieszka Gach, Bartosz Sakowicz, Mariusz Grzesiak, Hubert Huras, Tadeusz Pietrucha

**Affiliations:** ^1^Department of Medical Biotechnology, Medical University of Lodz, Łódź, Poland; ^2^Department of Obstetrics, Perinatology and Gynecology, Polish Mother's Memorial Hospital Research Institute, Łódź, Poland; ^3^Department of Obstetrics and Perinatology, University Hospital in Kraków, Kraków, Poland; ^4^Department of Genetics, Polish Mother's Memorial Hospital-Research Institute, Łódź, Poland; ^5^Department of Microelectronics and Computer Science, Lodz University of Technology, Łódź, Poland

## Abstract

**Background:**

Metalloproteinases (MMPs) play a pivotal role during the process of trophoblast invasion and placentation. The appearance of five functional single-nucleotide polymorphisms (SNP) in the genes of the metalloproteinases most commonly implicated in the implantation process may influence the development of preeclampsia.

**Methods:**

Blood samples were collected from 86 mothers and 86 children after preeclampsia and 85 mothers and 85 children with uncomplicated pregnancies. The distribution of genotypes for −1607 1G/2G *MMP1*, −735 C/T *MMP2*, −1306 C/T *MMP2*, −1171 5A/6A *MMP3*, and −1562C/T *MMP9* polymorphisms was determined by RFLP-PCR.

**Results:**

The occurrence of 1G/1G *MMP1* or 5A/5A *MMP3* genotype in the mother or 1G/1G *MMP1* or 5A/6A *MMP3* genotype in the child is associated with preeclampsia development. Moreover, simultaneous maternal and fetal 1G/1G homozygosity increases the risk of preeclampsia development 2.39-fold and the set of maternal 5A/5A and fetal 5A/6A *MMP3* genotypes by over 4.5 times. No association between the carriage of studied *MMP2* or *MMP9* polymorphisms and the predisposition to preeclampsia was found.

**Conclusion:**

The maternal 1G/1G *MMP1* and 5A/5A *MMP3* and fetal 1G/1G *MMP1* and 5A/6A *MMP3* gene polymorphisms may be strong genetic markers of preeclampsia, occurring either individually or together.

## 1. Introduction

Primary preeclampsia is a multifactorial disease. According to the American College of Obstetricians and Gynecologists (ACOG) criteria, preeclampsia is defined as gestational hypertension typified by ≥140 mmHg systolic or ≥90 mm Hg diastolic blood pressure measured on at least two occasions, accompanied by proteinuria, manifested as ≥0.3 g in a 24-hour collection of urine or ≥1+ in occasional urine samples, noticed after 20 weeks of gestation [1, 2]. The International Society for the Study of Hypertension in Pregnancy (ISSHP) raised the proteinuria threshold to 2+ on a dipstick [1, 3]. However, in the absence of proteinuria, the new onset of hypertension combined with the maternal organ dysfunction (according to ACOG and ISSHP criteria) or uteroplacental dysfunction (according to ISSHP criteria) is sufficient to recognize preeclampsia [1–3]. Preeclampsia appears in 5 to 8% of pregnancies and it is also a leading cause of perinatal morbidity and mortality [4, 5].

Although the precise etiology of preeclampsia is unknown, it has been suggested that while both genetic and environmental factors influence its development, genetic factors have a greater influence, accounting for over half of preeclampsia liability cases [6, 7]. Studies indicate that daughters born after preeclamptic pregnancy may carry susceptibility genes inherited from their mother, which predispose them to preeclampsia development [8]. Paternal genes carried by the fetus are also responsible for triggering preeclampsia development, but this risk is lower than the risk associated with the carriage of maternal genes [8, 9].

One of the most probable pathomechanisms of PE development is connected with inadequate maternal blood flow in the placenta, caused by deficient spiral artery remodeling and shallow trophoblast invasion. The process of placentation and the development of embryonic tissue and the extraembryonic trophoblast depends on the genome, which consists of both paternal and maternal genes [6].

In physiological conditions, the invasion of the extravillous trophoblast involves the integrin heterodimers which recognize the laminin/collagen IV and fibronectin in the decidual extracellular matrix (ECM) [5]. The basement membrane proteins of a newly synthesized ECM play a significant role in the crosstalk between fetal and maternal surfaces. The process of trophoblast adhesion is followed by enzymatic degradation of the decidual extracellular matrix, which is mediated principally by matrix metalloproteinases (MMPs). Metalloproteinases belong to a large family of zinc-dependent endopeptidases that include collagenases (e.g., MMP1, MMP8, and MMP13), gelatinases (MMP2, MMP9), stromelysins (MMP3, MMP10), matrilysins (MMP7, MMP26), and transmembrane metalloproteinases types I and II. All are collectively capable of degrading extracellular matrix components, but numerous studies have particularly implicated MMP1, MMP2, MMP3, and MMP9 in the process of trophoblast implantation. The invading trophoblast cells are not alone in the process of metalloproteinase secretions: MMPs are also expressed by endometrial stromal cells, natural killer (NK) cells, and macrophages of the decidual area [10]. Immunohistochemical and biochemical studies performed on placentas and deciduas indicate an imbalance in the metalloproteinase secretions both by trophoblast and decidual cells in pregnancies complicated by preeclampsia [5, 11, 12].

Although the level of metalloproteinase gene expression is regulated by transcriptional and posttranscriptional mechanisms, the first one exerts the most influence. Most MMPs share common sequences in their promoter regions which create the binding sites for the following transcription factors: the activator protein- (AP-) 1 and -2 sites, the polyomavirus enhancer-A binding protein-3 (PEA3) site, the SP-1 site, the NF-*κ*B site, and the STAT site [13, 14]. Changes in the nucleotide sequences in the binding sites of transcription factors or transcription repressors alter the regulation of MMP gene expression and thus may have an impact on the improper placentation of the trophoblast into the uterine wall.

The aim of the present study was to determine whether the most common MMP1, MMP2, MMP3, and MMP9 gene polymorphisms in maternal and fetal genes are associated with preeclampsia. All selected polymorphisms: −1607 1G/2G *MMP1* (rs1799750), −735 C/T *MMP2* (rs2285053), −1306 C/T *MMP2* (rs243865), −1171 5A/6A *MMP3* (rs35068180) and −1562C/T *MMP9* (rs3918242) are functional, are localized in the promoter region of MMPs, are responsible for the regulation of MMP gene expression, and thus may affect the amount of protein synthesized. It also examines whether the set of maternal/fetal genotypes and whether the sex of the fetus and its genotype with regard to the studied polymorphisms influence the predisposition to preeclampsia development.

## 2. Material and Methods

### 2.1. Study and Control Groups

This is a case-control study including preeclamptic (*N* = 86) women and their children (*N* = 86) and those who are healthy with uncomplicated pregnancies (*N* = 85 mothers and *N* = 85 children). All members of the study group were Caucasians and residents in Poland. The study group included women, and their children, who developed maternal blood pressure higher than 140/90 mm·Hg (measured twice with an interval of at least six hours) with accompanied proteinuria (>300 mg per 24 hours or at least 2+ during a single urine test) after the 20th week of gestation. The study group included both patients with early (<34 weeks of pregnancy; *N* = 31) preeclampsia and late (≥34 weeks of pregnancy; *N* = 55) preeclampsia.

The remaining qualification criteria for examination were the same for the control and study groups: single pregnancy, no hypertension or diabetes mellitus before pregnancy, no gestational diabetes mellitus, no chromosomal aberration in the fetus, maternal body mass index (BMI) before pregnancy ≤ 30 kg/m^2^, and no other chronic maternal disorders. Informed consent was signed by each mother before delivery, and the study protocol was approved by the Medical University of Lodz Ethical Committee (number RNN/212/11/KE).

Whole venous blood was taken from all participants one to two hours before the beginning of delivery, whereas the umbilical blood was obtained immediately after birth. As all women in the preeclamptic group delivered the baby by caesarian section, due to clinical indications, the control samples were also collected from women who had delivered their baby by caesarian section. Indications for caesarian delivery in the control group were as follows: transverse or breech position of fetus, ophthalmological indications, orthopedic indications, or increased risk of uterine rupture because of previously performed cesarean delivery.

### 2.2. Method of Genotyping

DNA was isolated from maternal and fetal blood with the use of a commercial kit (Chemagen, Germany). The primers for PCR reaction were as follows: MMP1_F_5′-GAG TAT ATC TGC CAC TCC TTG AC-3′, MMP1_R_5′-CTT GGA TTG ATT TGA GAT AAG TCA TAg C-3′, MMP2_-735C/T_F_5′-GGT GGG TGC TTC CTT TAA CAT G-3′, MMP2_-735C/T_R_5′-GTA AAA TGA GGC TGA GAC CTG C-3′, MMP2_-1306C/T_F_5′-CTT CCT AGG CTG GTC CTT ACT G-3′, MMP2_-1306C/T_R_5′-GCT GAG ACC TGA AGA GCC A-3′, MMP3_F_5′-CAT TCC TTT GAT GGG GGG AAA gA-3′, MMP3_R_5′-GAA GGA ATT AGA GCT GCC ACA GC-3′, MMP9_F_5′-GCA GAT CAC TTG AGT CAG AAG TTC-3′, MMP9_R_5′-GGG AAA AAC CTG CTA ACA ACT C-3′. The following restriction endonucleases were used: *AluI*, *HinfI*, *BstX*I, *Tth111I*, and *SphI* (Thermo Fisher Scientific, USA). The products of digestion were analyzed on 6% polyacrylamide gel and dyed with ethidium bromide.

### 2.3. Statistical Analyses

Data analysis was performed using Statistica v12 (StatSoft, Tulsa, OK). The clinical and personal characteristics data was normally distributed and so was compared between groups using Student's *t*-test. The Hardy–Weinberg equilibrium was tested in maternal and fetal control groups. Nominal variable comparisons were performed using chi^2^ or Yates' corrected chi^2^ tests. Multivariate analysis was performed with backward stepwise logistic regression on variables found to be significant (*p* < 0.05) in univariate analyses. Odds ratios (OR) with 95% confidence intervals (95% CI) were calculated for significant parameters obtained by multivariate and univariate analyses.

Haplotype analysis (haplotype frequency estimation and linkage disequilibrium) was performed between the maternal study and control groups and between the group of fetuses born from preeclamptic and those born from noncomplicated pregnancies; online SNPStat software was used for this purpose (https://www.snpstats.net/snpstats/) [15]. For each haplotype onset, the cumulative frequency was calculated; in addition, the association between the most frequent haplotype onsets (regarded as a cumulative frequency for haplotype onset score below 0.9) and preeclampsia was also calculated by logistic regression in SNPStat software. The results of the logistic regression were presented as ORs and 95% CI and *p* values.

Moreover, the Generalized Multifactor Dimensionality Reduction (GMDR, v0.9; http://www.ssg.uab.edu/gmdr/) [16, 17] analysis was used to calculate the interaction among five studied polymorphisms (−1607 1G/2G *MMP1* (rs1799750), −735 C/T *MMP2* (rs2285053), −1306 C/T *MMP2* (rs243865), −1171 5A/6A *MMP3* (rs35068180), and −1562C/T *MMP9* (rs3918242)) in predicting of the occurrence of preeclampsia. Maximum testing balanced accuracy (TBA) and cross-validation consistency (CVC) were used to select the best interaction model, and the results of analyses were considered to be statistically significant at *p* < 0.05.

## 3. Results

### 3.1. Baseline of the Study and Control Population

A total of 171 cases: 86 mothers and 86 children after preeclampsia and 85 mothers and 85 children with uncomplicated pregnancies were enrolled to this study. The comparisons of the baseline laboratory parameters and the characteristics of the patients of the study and control groups are summarized in Table 1.

### 3.2. The Influence of Maternal and Fetal MMP Gene Polymorphisms on the Risk of Preeclampsia Development

A week of linkage disequilibrium between studied polymorphisms was observed (Table 2).

Univariate analyses of the studied polymorphisms of maternal and fetal genotypes revealed that the maternal homozygosity of *MMP1* 1G/1G and the maternal homozygosity of *MMP3* 5A/5A are more common among preeclamptic women. No associations between the occurrence of preeclampsia and the distribution of genotypes or alleles of studied maternal *MMP2* and *MMP9* gene polymorphisms were observed. The distribution of genotypes and alleles in the maternal study and control groups are presented in Table 3.

Univariate analyses related to distribution of genotypes and alleles of studied −1607 1G/2G *MMP1* (rs1799750), −735 C/T *MMP2* (rs2285053), −1306 C/T *MMP2* (rs243865), −1171 5A/6A *MMP3* (rs35068180), and −1562C/T *MMP9* (rs3918242) gene polymorphisms among preeclamptic and control children found that the homozygosity of *MMP1* 1G/1G dominates in the group of children born from preeclamptic pregnancies. Additionally, the univariate analyses revealed that fetal heterozygosity 5A/6A *MMP3* is significantly more frequent in the population of preeclamptic children. No associations between occurrence of preeclampsia and the distribution of genotypes or alleles of studied fetal *MMP2* and *MMP9* gene polymorphisms were observed. The distribution of genotypes and alleles in the fetal study and control groups is presented in Table 4.

The distribution of all studied genotypes in the maternal and fetal control groups was consistent with the Hardy–Weinberg equilibrium.

Significant clinical parameters and statistically relevant maternal and fetal genotypes were included in the multivariable analysis. The results of this analysis are presented in Table 5.

Because fetal sex differed significantly among preeclamptic and noncomplicated pregnancies, it was interesting whether there exists some relationships in distribution of MMP genotypes in respect to fetal sex among study and control groups. This analysis reveals that the 1G/1G genotype of *MMP1* gene was significantly higher in the group of female preeclamptic children whereas 2G/2G genotype of *MMP1* dominated in the group of female and male children born from noncomplicated pregnancies. Moreover, this analysis pointed out that the carriage of 5A/6A genotype by male children is associated to preeclampsia occurrence whereas male fetal homozygous 6A/6A of *MMP3* dominate in noncomplicated pregnancies (Table 6).

Test of haplotypes identified a lot of their sets; however, any differences in the frequency haplotypes between the study and control groups of women were observed (Table 7). The results of the frequency of haplotypes among children born from preeclamptic and noncomplicated pregnancies have shown that only one haplotype 1G-5A-C-C-T is associated with the elevated maternal risk of occurrence of preeclampsia (OR = 5.93, 95% CI 1.06–33.19; *p* = 0.044). No other differences for frequency of haplotypes in children group were observed (Table 7).

According to the GMDR selection model, the best model among the maternal group was a two-SNP interaction model, containing −1607 1G/2G *MMP1* (rs1799750) and −1171 5A/6A *MMP3* (rs35068180), with the maximum cross-validation consistency (CVC) of 10/10 and with the maximum testing balanced accuracy (TBA) = 64.25% and significance of the test *p* = 0.0107. The second was a three-SNP interaction model, containing −1607 1G/2G *MMP1* (rs1799750), −1171 5A/6A *MMP3* (rs35068180), and −1562C/T *MMP9* (rs3918242), with the maximum CVC (9/10), TBA = 61.97%, and *p* = 0.0107.

The GMDR analysis used to calculate the interaction among 5 studied polymorphisms in predicting preeclampsia based on the gene-gene interaction in the children group has shown that the best model is containing only −1607 1G/2G *MMP1* (rs1799750) nucleotide polymorphisms, with the CVC (10/10), TBA = 63.82%, and *p* = 0.0107. The second model was based on a two-SNP interaction model containing −1607 1G/2G *MMP1* (rs1799750) and −1171 5A/6A *MMP3* (rs35068180), with the maximum cross-validation consistency (CVC) of 4/10 and with the maximum testing balanced accuracy (TBA) = 57.91% and significance of the test *p* = 0.0107. The last model based on a three-SNP interaction model containing −1607 1G/2G *MMP1* (rs1799750), −1171 5A/6A *MMP3* (rs35068180), and −1562C/T *MMP9* (rs3918242), with the CVC (7/10) and TBA = 53.92% was not statistically significant having *p* = 0.1719.

Because in all statistical tests we observed the contribution of 1G/2G *MMP1* and 5A/6A *MMP3* polymorphisms in the development of preeclampsia, we conduct the additional analyses focusing on simultaneous carriage of maternal (*MMP1* and *MMP3*) or fetal (*MMP1* and *MMP3*) or both maternal and fetal *MMP1* and *MMP3* gene polymorphisms and the predisposition to preeclampsia. These analyses revealed that simultaneously maternal homozygosity in 1G/1G *MMP1* and 5A/5A *MMP3* increases the risk of preeclampsia development over 10-fold (OR = 10.22, 95% CI 2.28–45.8; *p* < 0.01). We also observed that the fetal genotype elevates the maternal risk of preeclampsia development (OR = 6.54, 95% CI 2.13–20.01; *p* < 0.01) for simultaneous carriers of 1G/1G *MMP1* and 5A/6A *MMP3* polymorphisms. Additionally, we observed that simultaneous maternal and fetal 1G/1G homozygosity increases the risk of preeclampsia development 2.39-fold (OR = 2.39, 95% CI 1.01–5.65), whereas the set of maternal 5A/5A and fetal 5A/6A *MMP3* genotypes elevates the risk of occurrence of the studied disease 4.57 times (OR = 4.57, 95% CI 1.85–11.26).

## 4. Discussion

The regulation of metalloproteinase (MMP) secretion in the maternal-fetal interface plays a pivotal role in the process of trophoblast invasion and placentation. MMPs are known to be secreted by trophoblastic cells, and studies suggest that they are also expressed by maternal endometrial stromal cells and immunological cells which migrate into the uterus during perimenstrual and early pregnancy phases [10, 18]. Disturbances in metalloproteinase secretion by trophoblast or maternal cells may impair the process of trophoblastic invasion and placentation, thus inducing pregnancy-related disease such as preeclampsia or IUGR development.

The present study examines whether the occurrence of single-nucleotide polymorphisms (SNP) in the *MMP1*, *MMP2*, *MMP3*, and *MMP9* genes is related to the outcome of preeclampsia. Our results show that the maternal 1G/1G *MMP1* and 5A/5A *MMP3* gene polymorphisms occur significantly more frequently in preeclamptic cases. No relationship was observed between the maternal or fetal polymorphisms of *MMP2* and *MMP9* genes and adverse pregnancy outcome. The −1562C/T *MMP9*, −735 C/T, and −1306 C/T *MMP2* polymorphisms were localized in the promoter region of metalloproteinase genes, and numerous studies have implicated them in the regulation of gene expression. As the cytosine form of the −1562C/T *MMP9* polymorphism creates a binding site for a transcription repressor protein, the thymidine variant should induce higher relative *MMP9* gene expression: this has been confirmed on MALU cells [19]. Immunohistochemical experiments comparing preeclamptic women with age-matched controls based on placental sections of decidual cells and adjacent interstitial trophoblasts revealed that an elevated level of MMP9 is characteristic for gestation complicated by hypertension and proteinuria [20]. High MMP9 activity has also been observed in the umbilical cord plasma of preeclamptic newborns [21]. A similar observation of the elevated level of MMP9 has also been reported in the serum of preeclamptic women [22].

The studies conducted by Poon et al. [22], Galewska et al. [21], and Lockwood et al. [20] may suggest that the carriage of the TT genotype of −1562C/T polymorphism by expecting women could be related to the development of preeclampsia. However, study of Coolman et al. [23] on population of Dutch women whose gestation proceeded with or without preeclampsia pointed that the carriers of T allele had a lower risk of development of hypertension and proteinuria during pregnancy [23]. Our present study points at the lack of significant discrepancy in the distribution of the TT genotype and T allele between the study and control groups. The discrepancy between our study and the study of Coolman et al. [23] may be related to the population differences between Polish and Dutch preeclamptic women or various criteria of qualification of women on both studies. Results of our study are in agreement with those of Palei et al. [24], who noted that the carriage of the T allele at −1562C/T *MMP9* is not related with the development of preeclampsia.

The most comprehensively described metalloproteinase other than MMP9 is MMP2, which has been strongly implicated in the process of trophoblast invasion. The present study examines two functional SNPs (−735 C/T and −1306 C/T) localized in the promoter region of the *MMP2* gene which are related to the regulation of *MMP2* gene expression. The occurrence of thymidine at the −735 and −1306 positions negates the binding site for transcription factor Sp-1, thus decreasing the promoter activity of *MMP2* [25, 26]. An elevated MMP2 level was observed in the blood of preeclamptic women [27]. In addition, Palei et al. [28] found −1306C/T and −735C/T *MMP2* SNPs to be associated with elevated MMP2 levels in the plasma of preeclamptic Brazilian women and mothers whose pregnancies were accompanied by hypertension; however, they did not find any significant differences in the genotype or allele frequency distributions for the two SNPs when hypertensive or preeclamptic patients were compared with healthy cases [28]. These observations, in respect to −1306C/T polymorphism, were confirmed by Leonardo et al. among a Brazilian population [29]. Results of our study in respect to −1306C/T and −735C/T *MMP2* gene polymorphisms did not identify any significant relationship for genotype nor for allele distribution between preeclamptic and noncomplicated mothers or between their children; thus, they are in agreement with the results of Palei et al. [28] and Leonardo et al. [29].

Our univariate analyses found that homozygous 1G/1G of *MMP1* is more frequent in both mother and child in the preeclamptic group. The 1G/1G genotype is related to a lower tendency for *MMP1* gene expression [30]. Some studies note that the reduction of MMP1 levels in the umbilical cord blood, placenta, and decidua is characteristic for patients whose gestation is accompanied by preeclampsia [31, 32]. Interestingly, our univariate analysis comparing the male and female children of the study and control groups found almost a five times greater likelihood of preeclampsia in the mother of female fetuses whose *MMP1* genotype is 1G/1G (OR = 4.58, 95% CI 1.43–14.67; *p* < 0.02): these female children may have a higher tendency to develop preeclampsia if they later become pregnant. Moreover, simultaneous carriage of 1G/1G polymorphisms by mother and fetus appears to more than double the risk of occurrence of preeclampsia (OR = 2.39, 95% CI 1.01–5.65). Our findings do not confirm those of Jurajda et al. [33], who did not find any significant association between 1G/2G *MMP1* gene polymorphisms and pregnancy hypertension in a Caucasian population; however, the study of Jurajda et al. included women with recognized preeclampsia, eclampsia, and chronic hypertension, while patients with chronic hypertension were excluded in the present study.

Our findings also associate carriage of *MMP3* 5A/6A with the appearance of preeclampsia. The carriage of the 5A/5A genotype by the mother or the 5A/6A genotype by her child appears to increase the likelihood of the development of preeclampsia by over two times: OR = 2.76 for 5A/5A (95% CI 1.17–6.49; *p* < 0.05) and OR = 2.44 for 5A/6A (95% CI 1.16–5.13; *p* < 0.05). When both mother and fetus are carriers of both 5A/5A and 5A/6A *MMP3* polymorphisms, respectively, the risk of occurrence of preeclampsia is doubled OR = 4.57 (95% CI 1.85–11.26). Our observations may explain the results of other studies, which note that the MMP3 level is significantly higher in the decidual cells of preeclamptic mothers than gestational age-matched controls [5, 20]. The 5A variant is associated with greater promoter activity than variant 6A. Interestingly, the majority of children born from mothers who developed preeclampsia were heterozygous 5A/6A: a genotype associated with moderate MMP3 level. This may explain various observations according to MMP3 levels and preeclampsia presented in numerous studies. GDS3467 and GDS2080 studies included in the GEO Profile database demonstrate a lack of association between placental *MMP3* gene expression levels and preeclampsia, whereas others show downregulation of MMP3 levels in preeclamptic fetal origin cells and in preeclamptic extravillous trophoblast cells [12, 34].

One potential limitation of this study may be its relatively small number size: 86 mothers and 86 children in the study group and 85 mothers and 85 children in the controls. The power analyses (estimated using the G^∗^Power tool [35, 36]; data not included in Results) for the chi^2^ tests which yielded significant results indicated quite high power (between 80 and 98%): the chi^2^ tests for maternal 1G/1G *MMP1* and 1G/2G *MMP1* gene polymorphisms were 82% and 95%, respectively; the chi^2^ test for maternal 5A/5A *MMP3* was 93%; the chi^2^ tests for fetal 1G/1G and 2G/2G were 89% and 98%, respectively. Conversely, the power analyses for chi^2^ tests which did not yield significant results presented relatively low power (below 80%) and for this reason, the results could be inconclusive. This low power may be attributed to the relatively small number of cases qualified to the study and/or the rare appearance of minor variants for −735 C/T *MMP2* (rs2285053), −1306 C/T *MMP2* (rs243865), and −1562C/T *MMP9* (rs3918242) gene polymorphisms observed in both the study and control groups.

## 5. Conclusions

To summarize, our findings suggest that maternal SNPs −1607 1G/1G *MMP1* and −1171 5A/5A *MMP3* are independency important genetic factors associated with the occurrence of preeclampsia in Polish population. The simultaneous carriage of 1G/1G *MMP1* and 5A/5A *MMP3* polymorphisms by mother increases over 10-fold her risk to preeclampsia development. The fetal set of 1G/1G *MMP1*- and 5A/6A *MMP3*-studied polymorphisms also contributes to maternal predisposition of occurrence of preeclampsia OR = 6.54 (95% CI 2.13–20.01, *p* < 0.01). Additionally, simultaneous maternal and fetal 1G/1G homozygosity increases the risk of preeclampsia development 2.39-fold, whereas the set of maternal 5A/5A and fetal 5A/6A *MMP3* genotypes elevated the risk of occurrence of the studied disease by over 4.5 times. Moreover, our study confirms the observations of other researchers that the promoter polymorphisms of the *MMP2* and *MMP9* genes exert an insignificant effect on preeclampsia development.

## Figures and Tables

**(a):** 

Clinical data	Maternal preeclamptic group (*N* = 86)	Maternal control group (*N* = 85)	*p* value
Age of women (years)^1^	30.7 ± 6.2	32.0 ± 4.3	0.129
BMI (kg/m^2^)^1^	26 ± 3.1	25 ± 4.3	<0.05
WBC (10^3^/*μ*l)^1^	10.6 ± 2.3	10.7 ± 2.3	0.712
RBC (10^6^/*μ*l)^1^	4.1 ± 0.5	4.1 ± 0.4	0.648
HB (g/dl)^1^	12.2 ± 1.4	12.4 ± 1.1	0.474
HCT (%)^1^	35.7 ± 3.4	36.2 ± 2.5	0.313
MCV (fl)^1^	85.4 ± 6.5	87.1 ± 5.2	0.071
MCHC (g/dl)^1^	34.3 ± 2.6	33.8 ± 1.7	0.126
PLT (10^3^/*μ*l)^1^	203.4 ± 60.5	220.4 ± 54.2	0.057
History of miscarriage *n* (%)^2^	16 (19%)	13 (15%)	0.564
Week of delivery for early PE^1^ (*N* = 31)	31.6 ± 2.0	38.5 ± 1.0	<0.0001
Week of delivery for late PE^1^ (*N*=55)	37.6 ± 2.0	38.5 ± 1.0	<0.01

**(b):** 

Clinical data	Fetal preeclamptic group (*N* = 86)	Fetal control group (*N* = 85)	*p* value
Baby weight (g)^1^	2469 ± 923	3407 ± 448	<0.001
Baby height (cm)^1^	49.0 ± 6.4	54.1 ± 2.8	<0.001
Apgar score—1 minute^1^	8.6 ± 1.4	9.4 ± 0.8	<0.001
Baby sex (son), *N* (%)^2^	52 (60%)	38 (45%)	<0.05

^1^The values are presented as mean ± standard deviation. For data analysis, Student's *t*-test was used. ^2^For data analysis, the chi-squared test was used; BMI: body mass index; WBC: white blood cells; RBC: red blood cells; HB: haemoglobin concentration; HCT: haematocrit; MCV: mean corpuscular volume; MCHC: mean corpuscular haemoglobin concentration; PLT: platelets; kg/m^2^: kilograms/meter square; *μ*l: microlitre; g/dl: grams/decilitre; %: percent; fl: femtolitre; g: grams; cm: centimetre; *N*: number of cases.

**Table 2:** Linkage disequilibrium between studied polymorphisms.

	MMP3 rs35068180 (*D*')	MMP9 rs3918242 (*D*')	MMP2 rs2285053 (*D*')	MMP2 rs243865 (*D*')
MMP1 rs1799750 (*D*')	0.22	0.17	0.03	0.03
MMP3 rs35068180 (*D*')		0.30	0.27	0.07
MMP9 rs3918242 (*D*')			0.31	0.07
MMP2 rs2285053 (*D*')				0.25

*D*'is equal to *D* scaled in [−1,1] range where *D* is the deviation between the expected haplotype frequency and the observed frequency.

**Table 3:** Comparison of maternal genotypes between the preeclamptic and study groups.

Maternal genotypes and alleles	PE group *N* = 86 *N* (%)	Control group *N* = 85 *N* (%)	*p* ^a^	pHWE^b^
−1607 1G/2G *MMP1* (rs1799750)				
1G/1G	30 (35%)	14 (16%)	<0.01	0.712
1G/2G	22 (25%)	43 (51%)	<0.01	
2G/2G	34 (40%)	28 (33%)	0.369	
1G	82 (48%)	71 (42%)	0.272	
2G	90 (52%)	99 (58%)		

−1171 5A/6A *MMP3* (rs35068180)				
5A/5A	32 (37%)	12 (14%)	<0.01	0.585
5A/6A	31 (36%)	43 (51%)	0.055	
6A/6A	23 (27%)	30 (35%)	0.226	
5A	95 (55%)	67 (39%)	<0.01	
6A	77 (45%)	103 (61%)		

−1562C/T *MMP9* (rs3918242)				
CC	61 (71%)	57 (67%)	0.584	0.629
CT	25 (29%)	26 (31%)	0.828	
TT	0 (0%)	2 (2%)	0.472	
C	147 (85%)	140 (82%)	0.433	
T	25 (15%)	30 (18%)		

−735 C/T *MMP2* (rs2285053)				
CC	67 (78%)	66 (78%)	0.967	0.481
CT	19 (22%)	17 (20%)	0.737	
TT	0 (0%)	2 (2%)	0.472	
C	153 (89%)	149 (88%)	0.707	
T	19 (11%)	21 (12%)		

−1306 C/T *MMP2* (rs243865)				
CC	52 (60%)	49 (58%)	0.708	0.636
CT	30 (35%)	30 (35%)	0.955	
TT	4 (5%)	6 (7%)	0.730	
C	134 (78%)	128 (75%)	0.568	
T	38 (22%)	42 (25%)		

^a^For *p* value analysis, the chi^2^ or Yates' corrected chi^2^ tests were used; ^b^HWE: Hardy–Weinberg equilibrium.

**Table 4:** Comparison of genotypes between children born from preeclamptic and noncomplicated pregnancies.

Fetal genotypes and alleles	PE group *N* = 86 *N* (%)	Control group *N* = 85 *N* (%)	*p* ^a^	pHWE^b^
−1607 1G/2G *MMP1* (rs1799750)				
1G/1G	30 (35%)	12 (14%)	<0.01	0.503
1G/2G	43 (50%)	36 (42%)	0.316	
2G/2G	13 (15%)	37 (44%)	<0.001	
1G	103 (60%)	60 (35%)	<0.0001	
2G	69 (40%)	110 (65%)		

−1171 5A/6A *MMP3* (rs35068180)				
5A/5A	12 (14%)	17 (20%)	0.291	0.841
5A/6A	62 (72%)	41 (48%)	<0.01	
6A/6A	12 (14%)	27 (32%)	<0.01	
5A	86 (50%)	75 (44%)	0.276	
6A	86 (50%)	95 (56%)		

−1562C/T *MMP9* (rs3918242)				
CC	67 (78%)	61 (72%)	0.354	0.487
CT	19 (22%)	21 (25%)	0.687	
TT	0 (0%)	3 (3%)	0.240	
C	153 (89%)	143 (84%)	0.190	
T	19 (11%)	27 (16%)		

−735 C/T *MMP2* (rs2285053)				
CC	66 (77%)	69 (81%)	0.477	0.857
CT	18 (21%)	15 (18%)	0.586	
TT	2 (2%)	1 (1%)	0.992	
C	150 (87%)	153 (90%)	0.417	
T	22 (13%)	17 (10%)		

−1306 C/T *MMP2* (rs243865)				
CC	50 (58%)	48 (56%)	0.825	0.304
CT	27 (31%)	34 (40%)	0.240	
TT	9 (11%)	3 (4%)	0.140	
C	127 (74%)	130 (76%)	0.573	
T	45 (26%)	40 (24%)		

^a^For *p* value analysis, the chi^2^ or Yates' corrected chi^2^ tests were used; ^b^HWE: Hardy–Weinberg equilibrium.

**Table 5:** Multivariable analysis of clinical and genetic factors and predisposition to preeclampsia.

Parameters	OR	95% CI		*p*
BMI	1.13	1.02	1.23	<0.05
Maternal 1G/2G *MMP1* genotype	0.33	0.15	0.69	<0.01
Maternal 5A/5A *MMP3* genotype	2.76	1.17	6.49	<0.05
Fetal 5A/6A *MMP3* genotype	2.44	1.16	5.13	<0.05
Fetal 2G/2G *MMP1* genotype	0.31	0.14	0.70	<0.01

**Table 6:** Distribution of genotypes of studied polymorphisms among female and male preeclamptic and control groups.

Genotypes	Female PE children *N* = 34	Female control children *N* = 47	*p* ^a^	OR (95% CI)	Male PE children *N* = 52	Male control children *N* = 38	*p* ^a^	OR (95% CI)
−1607 1G/2G *MMP1* (rs1799750)								
1G/1G	12 (35%)	5 (10%)	<0.05	4.58 (1.43–14.67)	18 (35%)	7 (18%)	0.085	NS^b^
1G/2G	15 (44%)	21 (45%)	0.959	NS^b^	28 (54%)	15 (39%)	0.176	NS^b^
2G/2G	7 (21%)	21 (45%)	<0.05	0.32 (0.12–0.88)	6 (12%)	16 (42%)	<0.01	0.18 (0.06–0.52)

−1171 5A/6A *MMP3* (rs35068180)								
5A/5A	4 (12%)	9 (19%)	0.557	NS^b^	8 (15%)	8 (21%)	0.678	NS^b^
5A/6A	25 (73%)	26 (55%)	0.091	NS^b^	37 (71%)	15 (39%)	<0.01	3.78 (1.56–9.16)
6A/6A	5 (15%)	12 (26%)	0.366	NS^b^	7 (13%)	15 (39%)	<0.01	0.24 (0.08–0.67)

−1562C/T *MMP9* (rs3918242)								
CC	29 (85%)	32 (68%)	0.131	NS^b^	38 (73%)	29 (76%)	0.918	NS^b^
CT	5 (15%)	14 (30%)	0.188	NS^b^	14 (37%)	7 (18%)	0.490	NS^b^
TT	0 (0%)	1 (2%)	0.870	NS^b^	0 (0%)	2 (5%)	0.343	NS^b^

−735 C/T *MMP2* (rs2285053)								
CC	25 (73%)	35 (74%)	0.872	NS^b^	41 (79%)	34 (89%)	0.294	NS^b^
CT	8 (24%)	11 (23%)	0.801	NS^b^	10 (19%)	4 (11%)	0.406	NS^b^
TT	1 (3%)	1 (2%)	0.622	NS^b^	1 (2%)	0 (0%)	0.874	NS^b^

−1306 C/T *MMP2* (rs243865)								
CC	25 (73%)	28 (60%)	0.189	NS^b^	25 (48%)	20 (53%)	0.669	NS^b^
CT	8 (24%)	19 (40%)	0.107	NS^b^	19 (37%)	15 (39%)	0.777	NS^b^
TT	1 (3%)	0 (0%)	0.870	NS^b^	8 (15%)	3 (8%)	0.436	NS^b^

^a^For *p* value analysis, the chi^2^ or Yates' corrected chi^2^ tests were used; ^b^NS: not significant.

**Table 7:** Haplotype association with the predisposition to preeclampsia.

	Haplotype association with preeclampsia occurrence						
	MMP1 rs1799750	MMP3 rs35068180	MMP9 rs3918242	MMP2 rs2285053	MMP2 rs243865	OR (95% Cl)	*p* value
Maternal haplotype set							
(1)	2G	6A	C	C	C	1	—
(2)	1G	5A	C	C	C	0.78 (0.29–2.12)	0.63
(3)	2G	5A	C	C	C	2.90 (0.92–9.17)	0.072
(4)	1G	6A	C	C	C	2.64 (0.77–9.02)	0.12
(5)	2G	6A	C	C	T	0.63 (0.18–2.21)	0.47
(6)	2G	6A	C	C	C	1.66 (0.47–5.83)	0.43

Fetal haplotype set							
(1)	2G	6A	C	C	C	1	—
(2)	1G	5A	C	C	C	0.88 (0.28–2.75)	0.82
(3)	2G	5A	C	C	C	0.36 (0.09–1.48)	0.16
(4)	1G	6A	C	C	C	0.99 (0.28–3.44)	0.98
(5)	2G	6A	C	C	T	0.37 (0.08–1.77)	0.21
(6)	1G	6A	C	C	T	2.59 (0.61–11.09)	0.2
(7)	1G	5A	C	T	C	2.34 (0.59–9.29)	0.23
(8)	1G	5A	C	C	T	5.93 (1.06–33.19)	<0.05
(9)	2G	5A	T	C	C	0.98 (0.16–5.93)	0.98

OR: odds ratio; CI: confidence interval; result is statistically significant for *p* value < 0.05.
